# Sleep dissatisfaction and insufficient sleep duration in the Italian population

**DOI:** 10.1038/s41598-020-72612-4

**Published:** 2020-10-21

**Authors:** Nirosha Elsem Varghese, Alessandra Lugo, Simone Ghislandi, Paolo Colombo, Roberta Pacifici, Silvano Gallus

**Affiliations:** 1 Centre for Health and Social Care Management (CERGAS), SDA Bocconi School of Management, Bocconi University, Via Röntgen 1, 20136 Milan, Italy; 2 Laboratory of Lifestyle Epidemiology, Department of Environmental Health Sciences, Istituto di Ricerche Farmacologiche Mario Negri IRCCS, Via Mario Negri 2, 20156 Milan, Italy; 3 Istituto DOXA, Worldwide Independent Network/Gallup International Association (WIN/GIA), Via Panizza 7, 20144 Milan, Italy; 4 National Centre on Addiction and Doping, Istituto Superiore di Sanità, Viale Regina Elena, 299, 00161 Rome, Italy

**Keywords:** Epidemiology, Risk factors

## Abstract

To investigate the prevalence and possible determinants of sleep quality and quantity, we used data from a cross-sectional study conducted in 2019 on a sample of 3120 subjects, representative of the general Italian adult population. Sleep dissatisfaction was reported by 14.2% and insufficient sleep (duration) by 29.5% of adults. Sleep dissatisfaction and insufficient sleep were directly related with age (*p* for trend < 0.001), and inversely related with socioeconomic class (*p* for trend < 0.001) and income (*p* for trend < 0.001). Sleep dissatisfaction was higher among women (odds ratio, OR 1.30; 95% confidence interval, CI 1.05–1.60). Insufficient sleep was inversely related to education (*p* for trend < 0.001) and more frequent in current compared to never smokers (OR 1.32; 95% CI 1.08–1.61). Sleep dissatisfaction was higher among divorced/separated compared with married subjects (OR 1.75; 95% CI 1.20–2.58) and lower among subjects living with children aged 0–14 years (OR 0.48, 95% CI 0.33–0.70). Pet owners more frequently had sleep dissatisfaction (OR 1.35, 95% CI 1.08–1.68) and insufficient sleep (OR 1.46, 95% CI 1.23–1.73). In Italy, self-perceived sleep problems appear to be increasing. Sleep problems can contribute to aggravating health disparities in the society. The unfavourable relationship with pets (and the favourable ones with children) should be confirmed by longitudinal studies.

## Introduction

An adequate and healthy sleep is required for a good quality of life, better productivity and wellbeing^1^. Insufficient sleep has been found to be associated with physical health outcomes, including poor cardiovascular health, high blood pressure, insulin resistance and obesity^2–5^, mental health consequences, such as depression, suicidal and other self-harm behavior^6–8^, as well as overall mortality^2,9^. An estimated 7–9 h of total sleep is recommended for the adult population for healthy sleep^10^ whereas the US National Sleep Foundation (NSF) has put forward the four key indicators for good sleep quality to include (i) less difficulty in initiating sleep, (ii) sleeping more time while in bed, (iii) less than two instances of night waking and (iv) ability to go back to sleep within 20 min of a night awakening. More recently, sleep satisfaction was added as a further key indicator for good sleep quality^11,12^.

Given the importance of sleep on all aspects of health^2^, it is vital to monitor the prevalence of indicators of sleep quality and quantity at a population level and compare these estimates and trends within and between countries. Sleep quality can be measured either as a subjective rating regarding one’s satisfaction with sleep and/or as an index computed from several components related to sleep quality. Among others^13^, one widely used instrument for measuring sleep quality is the 24-item Pittsburgh Sleep Quality Index (PSQI)^14^, with translations and validations in several languages^15,16^. Efforts made to shorten the instrument using factor analyses showed that subjective sleep quality (i.e., sleep satisfaction) and sleep duration explained a significant portion of the variance of the PSQI score^16^.

To ensure objectivity of the measures, the self-completed questionnaires on sleep, when possible, are supplemented by objective measurements of sleep quality such as polysomnographic, actigraphy or movements captured using other phone apps or body wearables^17^. However, the obvious trade-off between higher accuracy of device supported measures versus the inexpensive and ease of large sample collection via self-reported information has led to population based epidemiological studies to use self-reported measures on sleep^16^.

In Italy, there are very few studies based on representative samples of the general population on self-reported sleep problems^18–20^. In particular, a cross-sectional study based on a sample of 3970 subjects, representative of the Italian general population found that 10.1% of Italian adults are dissatisfied with sleep^18^. The data used in this study dates back to 1997–1998. To our knowledge, no data after 2005 are available on sleep quality in Italy. Information on time spent sleeping is included in various surveys conducted by the Italian National Institute of Statistics. Using 2008–2009 time use data from ISTAT, Boffi and colleagues showed that in Italy, an average of 8 h and 17 min is spent for sleeping. Overall, they show that time spent sleeping has reduced in Italy, when compared to the past and to other European countries.

Given the paucity of data on the issue in the Italian population, we take advantage of a representative survey on Italian adults conducted in 2019 to assess the prevalence and determinants of sleep dissatisfaction and insufficient sleep (duration). To ensure comparability, we chose two questions on a subjective rating of overall sleep quality and actual sleep duration from the validated Italian version of the PSQI^15^.

## Methods

We used data from a survey, conducted by DOXA—the Italian branch of the Worldwide Independent Network/Gallup International Association (WIN/GIA)—and coordinated by the Italian National Institute of Health and by Mario Negri Institute^21^. Our analysis employs the data collected during February-April 2019 on a sample of 3120 subjects aged 15 years and over, representative of the general adult Italian population (52.4 million inhabitants in 2019), in terms of sex, age, area of residence and socioeconomic characteristics^22^.

Representative multistage sampling was applied to select participants. Briefly, the first stage involved the selection of 114 municipalities in all the 20 Italian regions, on the basis of region and size of municipalities. The second stage involved a random selection of an adequate number of electoral wards in each municipality, so that more or less affluent areas of each municipality were represented in the right proportions. In the third stage, individuals were randomly sampled from each electoral ward within strata of sex and age. A ‘quota’ method based on sex and age was used to select adolescents aged 15–17 years, since their names were not included in the electoral rolls. Finally, the data processing also involved generating statistical weights for each subject to ensure representativeness of the Italian population aged 15 years and older.

The interviews were conducted in Italian by ad hoc trained interviewers using a structured questionnaire in the context of a computer-assisted personal in-house (CAPI) interview.

Besides demographic (e.g., age, sex and marital status) and socioeconomic characteristics (e.g., geographic area, level of education, perceived socioeconomic level and self-reported family income), we collected information on smoking status, presence of children with various ages in the household and on pet ownership to indicate if the participants own one or more cats, one or more dogs, or one or more cats or dogs. Consistent with the validated Italian version of PSQI^15^, subjective evaluation of sleep quality was asked through the question: ‘During the past month, how would you rate your sleep quality overall?’. Possible answers were: (1) very good; (2) quite good; (3) quite bad (4) very bad. We defined individuals to be dissatisfied with sleep if participants evaluated their overall quality of sleep to be quite bad or bad^23^. Sleep quantity was reported in hours of sleep as a continuous measure and was assessed using the question: ‘During the past month, how many hours of actual sleep did you get at night?’. For our analysis, we defined ‘insufficient sleep’ as the ‘sleep duration that is likely too brief to meet physiologic needs’^24^. We used the cut-off of 6 h per night of sleep duration to disentangle those with insufficient sleep (≤ 6 h per night), as recommended by several panels and sleep societies^25^. According to the NSF recommendations on sleep time duration, sleeping 5–6 h may be appropriate (not recommended) for those who are 65 and above as the need for sleep decreases with age^10^. Therefore, we conduct a sensitivity check with a cutoff of ≤ 5 h of sleep per night defined as insufficient sleep duration for this age group.

To investigate determinants of sleep dissatisfaction and insufficient sleep, odds ratios (OR) and the corresponding 95% confidence intervals (CI) were estimated using multiple logistic regression models adjusted for sex, age, level of education, and geographic area. As a robustness check, we also repeat the analysis stratified by age groups (15–44, 45–64 and 65 and above) to examine if the determinants for sleep dissatisfaction and insufficient sleep duration vary by age. Our study is of exploratory nature and a significance level of 5% was chosen. The results were reported without any correction for multiplicity^26^. Statistical weights were used in all analyses to ensure representativeness of our sample. Statistical analysis was performed using SAS V.9.4 and Stata V.15 statistical software.

## Results

Of the 3120 Italian participants, 23.6% described their overall sleep quality as very good, 62.2% as quite good, 12.1% as quite bad and 2.1% as very bad. Mean sleep duration was 7.00 h per night (SD: 1.16; data not shown in tables).

Table 1 shows prevalence estimates for sleep dissatisfaction and insufficient sleep duration according to selected socioeconomic characteristics and lifestyle habits. Overall, 14.2% (95% CI 13.0–15.5%) of subjects reported having sleep dissatisfaction. The prevalence of subjects with sleep dissatisfaction was higher among women than men (OR 1.30; 95% CI 1.05–1.60), whereas no relationship with sex was observed for insufficient sleep. Both sleep dissatisfaction and insufficient sleep increased with age: compared to < 45 the ORs for sleep dissatisfaction were 2.81 (95% CI 2.12–3.71) for 45–64 years and 4.17 (95% CI 3.08–5.64; *p* for trend < 0.001) for ≥ 65 years; the corresponding ORs for insufficient sleep were 2.39 (95% CI 1.96–2.91) and 3.25 (95% CI 2.60–4.07; *p* for trend < 0.001), respectively. Whereas no statistically significant relation was observed between level of education and sleep dissatisfaction, insufficient sleep decreased with increasing of level of education (*p* for trend < 0.001). Both sleep dissatisfaction and insufficient sleep decreased with increasing socioeconomic class (*p* for trend < 0.001 for both measures of sleep) and income (*p* for trend < 0.001). No relation was observed between smoking status and sleep duration, but compared to never, current smoker had more frequently insufficient sleep (OR 1.32; 95% CI 1.08–1.61).

**Table 1:** Distribution of 3120 Italian participants aged ≥ 15 years, according to their sleep dissatisfaction and insufficient sleep, overall and by demographic and socio-economic characteristics, and smoking status. Corresponding odds ratios° (OR) and 95% confidence intervals (CI). Italy, 2019.

	N	Sleep dissatisfaction		Insufficient sleep duration	
		N (%)	OR (95% CI)	N (%)	OR (95% CI)
Total	3120	443 (14.2)		921 (29.5)	
**Sex**					
Male	1501	184 (12.3)	1.00^	449 (29.9)	1.00^
Female	1619	259 (16.0)	**1.30 (1.05–1.60)**	473 (29.2)	0.91 (0.78–1.07)
**Age**					
< 45 years	1281	82 (6.4)	1.00^	223 (17.4)	1.00^
45–64 years	1039	172 (16.5)	**2.81 (2.12–3.71)**	354 (34.0)	**2.39 (1.96–2.91)**
≥ 65 years	800	189 (23.6)	**4.17 (3.08–5.64)**	345 (43.1)	**3.25 (2.60–4.07)**
P for trend			**< 0.001**		**< 0.001**
**Education level**					
Low	1028	192 (18.7)	1.00^	395 (38.5)	1.00^
Intermediate	1579	202 (12.8)	0.93 (0.72–1.19)	420 (26.6)	**0.81 (0.67–0.99)**
High	513	50 (9.8)	0.73 (0.51–1.05)	106 (20.7)	**0.61 (0.47–0.80)**
P for trend			0.108		**< 0.001**
**Perceived socioeconomic class**					
Low	428	93 (21.8)	1.00^	193 (45.2)	1.00^
Intermediate	2294	310 (13.5)	**0.65 (0.49–0.87)**	628 (27.4)	**0.56 (0.44–0.70)**
High	398	40 (9.9)	**0.45 (0.29–0.70)**	100 (25.1)	**0.51 (0.37–0.71)**
P for trend			**< 0.001**		**< 0.001**
**Self-reported income**					
Low	1372	258 (18.8)	1.00^	515 (37.5)	1.00^
Intermediate	1004	118 (11.8)	**0.64 (0.50–0.83)**	271 (27.0)	**0.74 (0.61–0.89)**
High	744	67 (9.0)	**0.51 (0.37–0.70)**	136 (18.2)	**0.49 (0.39–0.62)**
P for trend			**< 0.001**		**< 0.001**
**Smoking status**					
Never smoker	2055	287 (14.0)	1.00^	570 (27.8)	1.00^
Ex-smoker	378	62 (16.5)	0.92 (0.68–1.26)	125 (33.1)	0.93 (0.72–1.18)
Current smoker	687	94 (13.6)	1.10 (0.84–1.44)	226 (32.9)	**1.32 (1.08–1.61)**

°ORs were estimated using unconditional multiple logistic regression models after adjustment for age groups (< 25; 25–44; 45–64; ≥ 65 years), sex, level of education and geographic area. Estimates in bold are statistically significant at 0.05 level.

^Reference category.

Table 2 shows prevalence estimates, and corresponding ORs, for sleep dissatisfaction and insufficient sleep by marital status, presence of children in household and ownership of pets. Compared to married, divorced or separated subjects had more frequently sleep dissatisfaction (OR 1.75; 95% CI 1.20–2.58), but not insufficient sleep. Subjects living with children aged 0–14 years (OR 0.48; 95% CI 0.33–0.70) had less frequently sleep dissatisfaction compared to participants without children. Results were similar in an additional specification when comparing participants living with no children aged 0–14 to those living with children below 5 but not between 6 and 14 years (OR 0.28; 95% CI 0.11–0.70) and those living with children between 6 and 14 years but not below 5 (OR 0.49; 95% CI 0.32–0.75). Overall, 32.1% of study participants had a pet (either a cat or a dog), 14.7% a cat and 23.2% a dog. Owning a pet (cat or dog) and owning both cats and dogs was directly related to both sleep dissatisfaction (OR 1.35; 95% CI 1.08–1.68 for a pet and OR 1.61; 95% CI 1.07–2.42 for both cats and dogs) and insufficient sleep (OR 1.46; 95% CI 1.23–1.73 for a pet and OR 2.12; 95% CI 1.52–2.95 for both cats and dogs). Additionally, owning dogs but not cats was directly related to insufficient sleep duration (OR 1.43, 95% CI 1.15–1.78).

**Table 2:** Distribution of 3120 Italian participants aged ≥ 15 years, according to their sleep dissatisfaction and insufficient sleep, by selected household characteristics. Corresponding odds ratios° (OR) and 95% confidence intervals (CI). Italy, 2019.

	N	Sleep dissatisfaction		Insufficient sleep duration	
		N (%)	OR (95% CI)	N (%)	OR (95% CI)
**Marital status**					
Married/cohabiting	1814	265 (14.6)	1.00^	575 (31.7)	1.00^
Single	879	68 (7.7)	1.15 (0.84–1.57)	171 (19.5)	1.09 (0.86–1.38)
Divorced/separated	172	41 (24)	**1.75 (1.20–2.58)**	57 (33.2)	1.09 (0.77–1.53)
Widowed	255	69 (26.9)	1.36 (0.96–1.92)	118 (46.4)	1.32 (0.98–1.78)
**Children 0–14 years**					
No	2492	405 (16.3)	1.00^	791 (31.8)	1.00^
Yes	628	38 (6.1)	**0.48 (0.33–0.70)**	130 (20.7)	0.80 (0.64–1.01)
**Children (age categories)**					
No children (0–14 years)	2492	405 (16.3)	1.00^	791 (31.8)	1.00^
Children below 5 but not between 6 and 14 years	150	5 (3.4)	**0.28 (0.11–0.70)**	25 (16.6)	0.66 (0.42–1.05)
Children between 6 and 14 but not below 5 years	414	26 (6.4)	**0.49 (0.32–0.75)**	89 (21.6)	0.81 (0.62–1.06)
Children below 5 and 6–14 years	64	7 (10.4)	0.97 (0.42–2.26)	16 (25.1)	1.11 (0.61–2.03)
**Pets**					
No	2118	279 (13.2)	1.00^	586 (27.7)	1.00^
Yes	1002	164 (16.4)	**1.35 (1.08–1.68)**	335 (33.4)	**1.46 (1.23–1.73)**
**Pets (cats/dogs)**					
No pets	2118	279 (13.2)	1.00^	586 (27.7)	1.00^
Cat but no dog	277	50 (17.9)	1.33 (0.94–1.88)	86 (31.1)	1.18 (0.89–1.57)
Dog but no cat	543	80 (14.8)	1.27 (0.96–1.68)	174 (32.0)	**1.43 (1.15–1.78)**
Cats and dogs	182	34 (18.8)	**1.61 (1.07–2.42)**	75 (41.1)	**2.12 (1.52–2.95)**

°ORs were estimated using unconditional multiple logistic regression models after adjustment for age groups (< 25; 25–44; 45–64; ≥ 65 years), sex, level of education and geographic area. Estimates in bold are statistically significant at 0.05 level.

^Reference category.

Tables S1 and S2 replicate the results in Table 1 stratified by age groups (i.e., 15–44, 45–64 and ≥ 65 years).

Tables S3 and S4 replicate the results in Table 2 stratified by age groups.

Table S5 reports findings for participants aged 65 and above when the cutoff of insufficient sleep duration is changed to ≤ 5 h, showing no changes when the cutoff for insufficient duration is changed from ≤ 6 to ≤ 5 h.

## Discussion

Our exploratory study shows that the prevalence estimates of sleep dissatisfaction and insufficient sleep in the Italian general adult population are 14% and 30%, respectively. Age and socio-economic characteristics are major determinants of sleep quality and quantity. Living with children is directly related with sleep quality whereas having pets is inversely related with both sleep quality and quantity.

Compared with the only representative study using our definition of sleep dissatisfaction, the sleep quality of Italian adults appears to be worsening from 10% in 1996–1997^18^ to 14% in 2019. Our estimates on the proportion of adults with insufficient sleep in Italy were higher than those observed in selected nationally representative surveys when comparable definitions were used, (i.e., actual sleep < 7 or ≤ 6 h per day), including Finland (14%) and Australia (17%)^27,28^, and similar to those observed in the USA (28–40%), with the higher estimates coming from studies using the Behavioral Risk Factors Surveillance System (BRFSS) and the National Health and Nutrition Examination Survey (NHANES) surveys respectively^29–33^.

We confirm the results of most, but not all^34–36^, previous studies in several European populations, including Italy^18^, showing higher subjective sleep dissatisfaction to be increasing with age^37–39^. Our analysis also showed insufficient sleep to be higher among older age groups, in agreement with current evidence^19,29,34,40^. In fact, previous studies have shown that sleep becomes more disturbed and lighter with age and this could be attributed to physiologic changes or decrease in time of melatonin production^29^.

Gender differences in sleep quality has been previously reported^41^. Our findings are consistent with past literature that shows women to report more sleep dissatisfaction compared to men^38,39,42,43^. These differences in sleep quality have been attributed to biological events in the life course, including hormonal influences or as a manifestation of sex differences in mental health which is more prevalent in women^44,45^. Moreover, women have been shown to express emotional concerns and bodily symptoms more readily in general compared to men^46^. However, we do not find significant results for the relationship between insufficient sleep and sex, in agreement with some^46,47^ but not all previous studies^48–50^.

In our analysis, the dimensions of socio-economic status (education, income and perceived socio-economic status) had a negative relationship with sleep dissatisfaction and insufficient sleep. Previous studies conducted in the US, European and Australian populations have consistently shown that lower socio-economic background, lower income families and low levels of education are risk factors for insufficient sleep and poor subjective sleep quality^24,28,43,51,52^. Previous studies using longitudinal data show that poverty ‘scars’, that is the ability of past exposure to poverty in reducing current life satisfaction even when an individual is out of poverty^53^. Given that sleep quality and quantity are significant predictors of life satisfaction, the scarring effects of poverty may take a toll on sleep health even when there is no current exposure to poverty^54^. Overall, the findings are in line with well documented evidence on social determinants of health including poverty and the role of sleep as a mediator between socioeconomic status (SES) and health^34,55,56^.

Previous findings are mixed with studies reporting either direct or non-significant relationship between being a current smoker and sleep problems^38,57^. However, we find a direct relationship between being a smoker and both sleep dissatisfaction and insufficient sleep among the younger population. When insufficient sleep is defined as ≤ 6 h, our results on smoking as a predictor of insufficient sleep are in line with findings from a study conducted among Australian adults aged 45–65 years^28^. In Finland, when insufficient sleep is defined as 1 h difference between self-reports of sleep need and sleep length, women who are current smokers were also more likely to report insufficient sleep^27^. A lower prevalence of sleep problems among former smokers in the older population could be attributed to an improved perception of sleep quality after quitting nicotine in addition to reduced need for sleep with increasing age^10^. It should also be noted that the mean age of former smokers is sixty years in our sample indicating large lifestyle changes around that age, which could be driving these results.

Several theoretical models explain the positive link between being married and health in general via sharing of resources to achieve better health outcomes^58^. A similar reasoning is applied to sleep outcomes with studies finding positive relationships between being married and better sleep outcomes including satisfaction with sleep^38,59–61^. In particular, epidemiological studies have shown sleep problems that extend beyond 10 weeks after marital separation to translate into worsening health outcomes in general^62^.

Prior literature shows the importance of not just presence or absence of children but also the age of the children in the household for sleep quality of the parents^63^. Contrary to previous findings, showing new parents to face up to 6 years of disrupted sleep^64^, our study finds no such effect. In fact, in our Italian population, we found an inverse relation between sleep dissatisfaction and living with children aged below 5 and 0–14 years. One explanation for these findings could be that our results are driven by older parents for a few possible reasons. In fact, older parents have been shown to have both more child rearing experience and positive attitude to parenting^65^. They are also relatively highly educated and earn more, therefore placing them in a position with fewer worries in life. They have fewer emotional and behavioral problems and the same is reflected in their offspring’s^66^. This gives an advantage to older parents in terms of both their own capabilities and that of the temperament of the offspring’s to enjoy parenthood better via either fewer sleep problems or better skills and resources to manage them.

Given the mean age of first time mothers in Italy is around 31 years^67^, one could approximate that participants driving this negative relationship to be second or third time parents and therefore strengthen our findings in line with the above explanation (the age group driving our results is 45–64 years). However, our reasoning can only be validated if the findings in the subgroup for younger parents are in an expected direction (positive) which is true for insufficient sleep. The same does not hold for sleep dissatisfaction which could be due to the fact that although respondents may sleep fewer hours than an average person, they may still be satisfied with their sleep. Among younger parents, life satisfaction increases following childbirth^68^ which may correspond to higher sleep satisfaction but not necessarily sleep duration.

Our findings could also be explained as an adaptation effect^69^ whereby living with a newborn baby will rescale your perception of a good quality sleep and after a time span the parent will perceive sleep quality much better than another person without a similar experience. However, the category ‘living with children below 5 years’ although not statistically significant also show a relationship in the same direction as other categories. Therefore, we need both studies with a larger sample size and information on children at even younger ages to see evidence of such an adaptation.

Furthermore, our findings on presence of children in the household are likely to be affected by potential confounding. In particular, there could be a omitted variable that is positively related to sleep problems and negatively related to presence of children in the household that could be confounding this relation. One could think of a variable like poor health of the respondent that may reduce fertility or fertility intentions^70^ and is also positively correlated to sleep problems^71^. Number of children is an endogenous variable^72^ and hence could also be subjected to reverse causality, which is sleep dissatisfaction and insufficient sleep duration predicting fewer children. Therefore, we require a good instrumental variable to tease out the true effect of presence of children in the household. Finally, our estimates could also be subjected to what is known as functional form approximation which arises due to fewer observations within a category of the variable^73^. In our case, the number of observations reporting having children in the household is far fewer than the control category (no children) and therefore the linear term with the negative sign could be the best approximation of the data. Our finding on children needs to be investigated and validated with datasets with larger samples and more information on other individual characteristics to make sure that our results are not driven by confounding or lack of power.

The presence of pets in the house can modify the sleep environment including odor, noise and temperature^74^. Our study shows that pet ownership in general or owning a cat or dog specifically increases the likelihood of being dissatisfied with sleep and having insufficient sleep duration. Although it is widely accepted in the medical community that pets should not be allowed into the bedrooms at night, evidence on the role of pet ownership on owner’s sleep quality is sparse. An Australian study based on a sample of 2036 adults found that participants who co-slept with their pets took longer to fall asleep, were more likely to wake up tired and disturbed during sleep by dog barking or other animal noises^75^. Our results on pet ownership were also partly consistent with two other recent empirical studies. A study based on 40 healthy adults with no sleep disorders and objective measurement of sleep showed that having a dog in the bedroom may disrupt the sleep of the owners only if the dog shares the bed^76^. Mein and Grant (2018) used elderly participants from the Whitehall II aged 59–79 years to show that dog owners were more likely to wake up more tired compared to non-dog owners^77^. However, our findings could also be biased given the cross-sectional nature of our data. For instance, pet owners could be a selected population who suffer more frequently from sleep problems or other health characteristics leading to pet selection effects^78,79^. Hence reverse causality cannot be excluded and should be confirmed using prospective longitudinal data. Given the mixed evidence, more recent empirical works point towards the need to prioritize the sleep of owners over loyalty to the pet. Moreover, health care workers should consider inquiring about pet ownership to patients with sleep concerns and discuss the pros and cons of having a pet as their sleep companion.

Our study has some limitations. Our analysis includes self-reported measures of sleep dissatisfaction and sleep duration^24^. Previous studies show that participants tend to overestimate self-reported measures of sleep. Second, our survey measures sleep duration in whole number hours which is less precise than sleep duration in fractions or minutes. But this does not hinder us in defining insufficient sleep at the cutoff ≤ 6 h. Also, the main sources of survey data in the US for sleep measures including BRFSS and NHANES uses sleep duration measured in whole number hours^29^. Third, previous studies show that prevalent health conditions such as cancer or lifestyle diseases such as obesity, heart disease or type-2 diabetes is either directly or indirectly related to sleep disruption through stress of disease burden^71^. Due to unavailability of information on any measure of health, our study could be exposed to omitted variability bias. However, controlling for smoking status could account for some measure of lifestyle which could be a proxy for current health status. Fourth , methodological drawbacks inherent to the cross-sectional study design, particularly problems of reverse causality, could not be ruled out. Finally, we performed several tests without taking into account multiple testing^26,80^. Therefore, the results from our exploratory study should be interpreted with caution, and should be confirmed by future studies. The strengths of our study include the relatively large sample size that allowed us to obtain estimates adjusted for multiple potential confounding factors, the sample representativeness of the general Italian adult population by age, sex and socioeconomic characteristics, and the use of validated and standardized assessment measures for sleep dissatisfaction and insufficient sleep.

Our study shows that the prevalence of sleep dissatisfaction and insufficient sleep in the Italian adult population is likely increasing and relatively high as compared to other European countries. Interventions to improve sleep quality and quantity should be tailored to the population with a disadvantage socioeconomic background. Our findings on pets (and children) should be further investigated. If confirmed by longitudinal data, they might have important implications from a public health perspective.

## Supplementary information

Supplementary information
