# Supplementary material for: Sleep dissatisfaction and insufficient sleep duration in the Italian population
Source: Sci Rep. 2020 Oct 21;10:17943. doi: 10.1038/s41598-020-72612-4 (PMC7578786; doi:10.1038/s41598-020-72612-4)
Supplement: Supplementary file 1 — Supplementary information [file 41598_2020_72612_MOESM1_ESM.docx]

**SLEEP DISSATISFACTION AND INSUFFICIENT SLEEP DURATION IN THE ITALIAN POPULATION**

Nirosha Elsem VARGHESE (MEc),^1^ Alessandra LUGO (PhD),^2^ Simone GHISLANDI (DPhil),^1^ Paolo COLOMBO (ScD),^3^ Roberta PACIFICI (ScD),^4^ Silvano GALLUS (ScD)^2*^

1) Centre for Health and Social Care Management (CERGAS), SDA Bocconi School of Management, Bocconi University, Via Röntgen 1, Milano 20136, Italy

2) Department of Environmental Health Sciences, Istituto di Ricerche Farmacologiche Mario Negri IRCCS, via Mario Negri 2, 20156 Milano, Italy

3) Istituto DOXA, Worldwide Independent Network/Gallup International Association (WIN/GIA), Via Panizza 7, 20144 Milano

4) National Centre on Addiction and Doping, Istituto Superiore di Sanità, Viale Regina Elena, 299, 00161 Roma, Italy

**Address correspondance to:**

Silvano Gallus, ScD

Laboratory of Lifestyle Epidemiology

Department of Environmental Health Sciences

Istituto di Ricerche Farmacologiche Mario Negri IRCCS

Via Mario Negri 2; 20156 Milan, Italy

Tel. +39 02 3901 4657

e-mail: silvano.gallus@marionegri.it

| **Table S1.** Distribution of 3120 Italian participants aged ≥15 years, according to their sleep dissatisfaction, stratified by age groups and by demographic and socio-economic characteristics, and smoking status. Corresponding odds ratios° (OR) and 95% confidence intervals (CI). Italy, 2019. | | | | | | | | | |
| --- | --- | --- | --- | --- | --- | --- | --- | --- | --- |
|  | Sleep dissatisfaction | | | | | | | | |
|  | 15-44 years | | | 45-64 years | | | ≥ 65 years | | |
|  | N | N (%) | OR (95% CI) | N | N (%) | OR (95% CI) | N | N (%) | OR (95% CI) |
| **Total** | 1281 | 83 (6.4) |  | 1039 | 172 (16.5) |  | 800 | 189 (23.6) |  |
|  |  |  |  |  |  |  |  |  |  |
| **Sex** |  |  |  |  |  |  |  |  |  |
| Male | 647 | 38 (5.8) | 1.00^ | 507 | 70 (13.9) | 1.00^ | 346 | 76 (22.1) | 1.00^ |
| Female | 634 | 45 (7.1) | 1.26 (0.80-1.97) | 532 | 101 (19.1) | **1.48 (1.06-2.06)** | 454 | 113 (24.8) | 1.16 (0.83-1.63) |
| **Education level** |  |  |  |  |  |  |  |  |  |
| Low | 198 | 13 (6.3) | 1.00^ | 294 | 49 (16.7) | 1.00^ | 536 | 130 (24.3) | 1.00^ |
| Intermediate | 786 | 54 (6.8) | 1.23 (0.64-2.34) | 588 | 99 (16.8) | 0.90 (0.61-1.34) | 205 | 50 (24.1) | 0.87 (0.59-1.28) |
| High | 297 | 16 (5.5) | 0.92 (0.43-1.97) | 157 | 24 (15.4) | 0.81 (0.47-1.40) | 59 | 9 (15.9) | 0.55 (0.26-1.14) |
| P for trend |  |  | 0.830 |  |  | 0.457 |  |  | 0.107 |
| **Perceived socioeconomic class** |  |  |  |  |  |  |  |  |  |
| Low | 137 | 10 (7.0) | 1.00^ | 111 | 20 (18.1) | 1.00^ | 179 | 64 (35.5) | 1.00^ |
| Intermediate | 990 | 67 (6.8) | 1.10 (0.52-2.31) | 757 | 129 (17.0) | 0.94 (0.55-1.61) | 547 | 115 (21.0) | **0.49 (0.33-0.72)** |
| High | 154 | 6 (3.8) | 0.58 (0.19-1.75) | 171 | 23 (13.4) | 0.69 (0.34-1.40) | 74 | 11 (14.8) | **0.28 (0.13-0.59)** |
| P for trend |  |  | 0.335 |  |  | 0.305 |  |  | **0.001** |
| **Self-reported income** |  |  |  |  |  |  |  |  |  |
| Low | 480 | 46 (9.6) | 1.00^ | 430 | 83 (19.3) | 1.00^ | 461 | 129 (27.9) | 1.00^ |
| Intermediate | 445 | 24 (5.3) | **0.57 (0.34-0.98)** | 329 | 50 (15.1) | 0.71 (0.48-1.06) | 230 | 45 (19.4) | **0.59 (0.39-0.90)** |
| High | 356 | 13 (3.6) | **0.39 (0.20-0.77)** | 280 | 39 (13.9) | **0.59 (0.37-0.93)** | 109 | 16 (14.4) | **0.46 (0.25-0.83)** |
| P for trend |  |  | **0.007** |  |  | **0.023** |  |  | **0.011** |
| **Smoking status** |  |  |  |  |  |  |  |  |  |
| Never smoker | 870 | 47 (5.4) | 1.00^ | 649 | 103 (15.9) | 1.00^ | 536 | 137 (25.6) | 1.00^ |
| Current smoker | 330 | 29 (8.8) | 1.63 (0.99-2.67) | 260 | 45 (17.1) | 1.11 (0.75-1.63) | 96 | 20 (20.8) | 0.71 (0.40-1.25) |
| Ex-smoker | 81 | 7 (8.1) | 1.56 (0.66-3.70) | 130 | 24 (18.6) | 1.22 (0.75-2.00) | 168 | 32 (18.9) | **0.60 (0.38-0.95)** |

° ORs were estimated using unconditional multiple logistic regression models after adjustment for sex, level of education and geographic area. Estimates in bold are statistically significant at 0.05 level.

^ Reference category.

| **Table S2.** Distribution of 3120 Italian participants aged ≥15 years, according to insufficient sleep duration, stratified by age groups and by demographic and socio-economic characteristics, and smoking status. Corresponding odds ratios° (OR) and 95% confidence intervals (CI). Italy, 2019. | | | | | | | | | |
| --- | --- | --- | --- | --- | --- | --- | --- | --- | --- |
|  | Insufficient sleep duration | | | | | | | | |
|  | 15-44 years | | | 45-64 years | | | ≥ 65 years | | |
|  | N | N (%) | OR (95% CI) | N | N (%) | OR (95% CI) | N | N (%) | OR (95% CI) |
| **Total** | 1281 | 223 (17.4) |  | 1039 | 354 (34.0) |  | 800 | 345 (43.1) |  |
|  |  |  |  |  |  |  |  |  |  |
| **Sex** |  |  |  |  |  |  |  |  |  |
| Male | 647 | 108 (16.7) | 1.00^ | 507 | 193 (38.1) | 1.00^ | 346 | 148 (42.8) | 1.00^ |
| Female | 634 | 115 (18.2) | 1.13 (0.84-1.51) | 532 | 161 (30.2) | **0.70 (0.54-0.91)** | 454 | 197 (43.4) | 1.02 (0.76-1.36) |
| **Education level** |  |  |  |  |  |  |  |  |  |
| Low | 198 | 39 (19.8) | 1.00^ | 294 | 106 (36.1) | 1.00^ | 536 | 250 (46.7) | 1.00^ |
| Intermediate | 786 | 139 (17.6) | 0.98 (0.66-1.47) | 588 | 201 (34.2) | 0.98 (0.72-1.33) | 205 | 80 (38.9) | **0.62 (0.44-0.87)** |
| High | 297 | 45 (15.3) | 0.78 (0.48-1.25) | 157 | 46 (29.5) | 0.79 (0.51-1.21) | 59 | 15 (24.8) | **0.33 (0.18-0.62)** |
| P for trend |  |  | 0.296 |  |  | 0.270 |  |  | **0.001** |
| **Perceived socioeconomic class** |  |  |  |  |  |  |  |  |  |
| Low | 137 | 31 (22.3) | 1.00^ | 111 | 48 (43.0) | 1.00^ | 179 | 115 (64.0) | 1.00^ |
| Intermediate | 990 | 166 (16.8) | 0.84 (0.53-1.34) | 757 | 251 (33.2) | 0.72 (0.47-1.09) | 547 | 211 (38.5) | **0.41 (0.29-0.60)** |
| High | 154 | 26 (17.1) | 0.85 (0.46-1.58) | 171 | 54 (31.9) | 0.72 (0.42-1.23) | 74 | 19 (25.9) | **0.21 (0.11-0.40)** |
| P for trend |  |  | 0.615 |  |  | 0.233 |  |  | **<0.001** |
| **Self-reported income** |  |  |  |  |  |  |  |  |  |
| Low | 480 | 108 (22.6) | 1.00^ | 430 | 169 (39.2) | 1.00^ | 461 | 238 (51.6) | 1.00^ |
| Intermediate | 445 | 76 (17.0) | 0.81 (0.58-1.14) | 329 | 108 (32.9) | 0.77 (0.57-1.06) | 230 | 87 (37.7) | **0.63 (0.44-0.89)** |
| High | 356 | 39 (11.0) | **0.52 (0.34-0.79)** | 280 | 76 (27.3) | **0.63 (0.44-0.90)** | 109 | 20 (18.5) | **0.26 (0.15-0.45)** |
| P for trend |  |  | **0.002** |  |  | **0.011** |  |  | **<0.001** |
| **Smoking status** |  |  |  |  |  |  |  |  |  |
| Never smoker | 870 | 124 (14.3) | 1.00^ | 649 | 214 (33.0) | 1.00^ | 536 | 232 (43.2) | 1.00^ |
| Current smoker | 330 | 86 (26.1) | **2.02 (1.47-2.79)** | 260 | 90 (34.4) | 1.00 (0.73-1.35) | 96 | 50 (51.9) | 1.39 (0.87-2.23) |
| Ex-smoker | 81 | 13 (15.8) | 1.09 (0.58-2.06) | 130 | 50 (38.1) | 1.19 (0.80-1.77) | 168 | 63 (37.5) | 0.72 (0.49-1.06) |

° ORs were estimated using unconditional multiple logistic regression models after adjustment for sex, level of education and geographic area. Estimates in bold are statistically significant at 0.05 level.

^ Reference category

| **Table S3.** Distribution of 3120 Italian participants aged ≥15 years, stratified by age groups and according to their sleep dissatisfaction, by selected household characteristics. Corresponding odds ratios° (OR) and 95% confidence intervals (CI). Italy, 2019. | | | | | | | | | |
| --- | --- | --- | --- | --- | --- | --- | --- | --- | --- |
|  | Sleep dissatisfaction | | | | | | | | |
|  | 15-44 years | | | 45-64 years | | | ≥ 65 years | | |
|  | N | N (%) | OR (95% CI) | N | N (%) | OR (95% CI) | N | N (%) | OR (95% CI) |
| **Total** | 1281 | 223 (17.4) |  | 1039 | 354 (34.0) |  | 800 | 345 (43.1) |  |
|  |  |  |  |  |  |  |  |  |  |
| **Marital status** |  |  |  |  |  |  |  |  |  |
| Married/Cohabiting | 545 | 42 (7.7) | 1.00^ | 782 | 125 (16.0) | 1.00^ | 487 | 98 (20.2) | 1.00^ |
| Single | 700 | 36 (5.1) | **0.61 (0.38-0.98)** | 136 | 21 (15.8) | 1.03 (0.62-1.71) | 43 | 11 (24.7) | 1.38 (0.65-2.91) |
| Divorced/Separated | 35 | 4 (12.8) | 1.89 (0.65-5.46) | 102 | 20 (19.9) | 1.26 (0.74-2.13) | 35 | 17 (47.1) | **4.06 (1.92-8.56)** |
| Widowed | 1 | 0 (0) | - | 19 | 5 (26.1) | 1.83 (0.64-5.20) | 235 | 64 (27.1) | **1.49 (1.01-2.20)** |
| **Children 0-14 years** |  |  |  |  |  |  |  |  |  |
| No | 835 | 58 (7.0) | 1.00^ | 873 | 160 (18.3) | 1.00^ | 784 | 187 (23.9) | 1.00^ |
| Yes | 446 | 24 (5.4) | 0.72 (0.43-1.18) | 166 | 12 (7.2) | **0.37 (0.20-0.68)** | 16 | 2 (11.5) | 0.55 (0.12-2.57) |
| **Children (Age categories)** |  |  |  |  |  |  |  |  |  |
| No children (0-14 years) | 835 | 58 (7.0) | 1.00^ | 873 | 160 (18.3) | 1.00^ | 784 | 187 (23.9) | 1.00^ |
| Children below 5 but not  between 6-14 years | 133 | 5 (3.4) | 0.46 (0.17-1.23) | 11 | 1 (5.8) | 0.29 (0.02-3.82) | 6 | 0 (0) | - |
| Children between 6-14  but not below 5 years | 257 | 13 (5.1) | 0.66 (0.35-1.23) | 147 | 11 (7.8) | **0.39 (0.21-0.74)** | 10 | 2 (18.1) | 0.78 (0.16-3.84) |
| Children below 5 and 6-14 years | 56 | 7 (12.0) | 1.62 (0.68-3.85) | 8 | 0 (0) | - | 0 | - | - |
| **Pets** |  |  |  |  |  |  |  |  |  |
| No | 870 | 53 (6.1) | 1.00^ | 668 | 98 (14.7) | 1.00^ | 580 | 128 (22.1) | 1.00^ |
| Yes | 411 | 29 (7.1) | 1.23 (0.76-1.97) | 371 | 74 (20.0) | 1.39 (0.99-1.94) | 220 | 61 (27.8) | 1.27 (0.88-1.82) |
| **Pets (Cats/Dogs)** |  |  |  |  |  |  |  |  |  |
| No pets | 870 | 53 (6.1) | 1.00^ | 668 | 98 (14.7) | 1.00^ | 580 | 128 (22.1) | 1.00^ |
| Cat but no dog | 95 | 7 (7.0) | 1.25 (0.54-2.92) | 90 | 17 (19.2) | 1.34 (0.75-2.37) | 92 | 26 (27.8) | 1.24 (0.75-2.07) |
| Dog but no cat | 249 | 18 (7.2) | 1.20 (0.68-2.10) | 207 | 37 (17.7) | 1.19 (0.78-1.81) | 87 | 26 (30.0) | 1.38 (0.83-2.32) |
| Cats and dogs | 67 | 5 (7.2) | 1.33 (0.50-3.52) | 74 | 20 (26.9) | **2.10 (1.20-3.69)** | 41 | 10 (23.1) | 1.07 (0.50-2.32) |

° ORs were estimated using unconditional multiple logistic regression models after adjustment for sex, level of education and geographic area. Estimates in bold are statistically significant at 0.05 level.

^ Reference category.

| **Table S4.** Distribution of 3120 Italian participants aged ≥15 years, stratified by age groups and according to insufficient sleep duration, by selected household characteristics. Corresponding odds ratios° (OR) and 95% confidence intervals (CI). Italy, 2019. | | | | | | | | | |
| --- | --- | --- | --- | --- | --- | --- | --- | --- | --- |
|  | Insufficient sleep duration | | | | | | | | |
|  | 15-44 years | | | 45-64 years | | | ≥ 65 years | | |
|  | N | N (%) | OR (95% CI) | N | N (%) | OR (95% CI) | N | N (%) | OR (95% CI) |
| **Total** | 1281 | 223 (17.4) |  | 1039 | 354 (34.0) |  | 800 | 345 (43.1) |  |
|  |  |  |  |  |  |  |  |  |  |
| **Marital status** |  |  |  |  |  |  |  |  |  |
| Married/Cohabiting | 545 | 118 (21.7) | 1.00^ | 782 | 259 (33.1) | 1.00^ | 487 | 198 (40.5) | 1.00^ |
| Single | 700 | 103 (14.7) | **0.58 (0.43-0.79)** | 136 | 53 (39.4) | 1.34 (0.91-1.96) | 43 | 15 (35.0) | 0.77 (0.39-1.51) |
| Divorced/Separated | 35 | 2 (5.9) | 0.24 (0.06-1.00) | 102 | 34 (33.5) | 1.07 (0.69-1.67) | 35 | 21 (59.5) | **3.02 (1.43-6.36)** |
| Widowed | 1 | 0 (0) | - | 19 | 7 (36.9) | 1.33 (0.52-3.43) | 235 | 111 (47.4) | 1.34 (0.95-1.89) |
| **Children 0-14 years** |  |  |  |  |  |  |  |  |  |
| No | 835 | 138 (16.5) | 1.00^ | 873 | 314 (36.0) | 1.00^ | 784 | 339 (43.3) | 1.00^ |
| Yes | 446 | 85 (19.1) | 1.15 (0.85-1.57) | 166 | 39 (23.7) | **0.55 (0.37-0.81)** | 16 | 6 (34.6) | 1.05 (0.37-3.03) |
| **Children (Age categories)** |  |  |  |  |  |  |  |  |  |
| No children (0-14 years) | 835 | 138 (16.5) | 1.00^ | 873 | 314 (36.0) | 1.00^ | 784 | 339 (43.3) | 1.00^ |
| Children below 5 but not  between 6-14 years | 133 | 21 (16.0) | 0.99 (0.60-1.64) | 11 | 2 (22.2) | 0.47 (0.11-2.00) | 6 | 1 (19.7) | 0.68 (0.09-5.26) |
| Children between 6-14  but not below 5 years | 257 | 50 (19.5) | 1.16 (0.81-1.67) | 147 | 35 (23.6) | **0.55 (0.36-0.83)** | 10 | 5 (43.1) | 1.27 (0.36-4.51) |
| Children below 5 and 6-14 years | 56 | 14 (24.6) | 1.54 (0.80-2.94) | 8 | 2 (28.3) | 0.64 (0.14-2.94) | 0 | - | - |
| **Pets** |  |  |  |  |  |  |  |  |  |
| No | 870 | 148 (17.1) | 1.00^ | 668 | 208 (31.1) | 1.00^ | 580 | 231 (39.7) | 1.00^ |
| Yes | 411 | 75 (18.2) | 1.14 (0.84-1.57) | 371 | 146 (39.3) | **1.49 (1.14-1.95)** | 220 | 114 (51.9) | **1.66 (1.20-2.30)** |
| **Pets (Cats/Dogs)** |  |  |  |  |  |  |  |  |  |
| No pets | 870 | 148 (17.1) | 1.00^ | 668 | 208 (31.1) | 1.00^ | 580 | 231 (39.7) | 1.00^ |
| Cat but no dog | 95 | 13 (13.9) | 0.85 (0.46-1.57) | 90 | 30 (33.5) | 1.22 (0.76-1.96) | 92 | 43 (46.5) | 1.30 (0.82-2.06) |
| Dog but no cat | 249 | 47 (18.9) | 1.17 (0.81-1.69) | 207 | 82 (39.5) | **1.46 (1.05-2.03)** | 87 | 45 (51.6) | **1.61 (1.00-2.59)** |
| Cats and dogs | 67 | 14 (21.5) | 1.52 (0.82-2.82) | 74 | 34 (46.1) | **1.98 (1.21-3.24)** | 41 | 27 (64.4) | **3.14 (1.56-6.34)** |

° ORs were estimated using unconditional multiple logistic regression models after adjustment for sex, level of education and geographic area. Estimates in bold are statistically significant at 0.05 level.

^ Reference category.

| **Table S5.** Distribution of 800 Italian participants aged ≥65 years, according to insufficient sleep duration (≤5 hours per night) and individual and other household characteristics. Corresponding odds ratios° (OR) and 95% confidence intervals (CI). Italy, 2019. | | | |
| --- | --- | --- | --- |
|  | **N** | **N (%)** | **OR (95% CI)** |
| **Total** | 800 | 144 (18.0) |  |
| **Sex** |  |  |  |
| Male | 346 | 52 (15.0) | 1.00^ |
| Female | 454 | 92 (20.2) | 1.42 (0.97-2.08) |
| **Education level** |  |  |  |
| Low | 536 | 105 (19.5) | 1.00^ |
| Intermediate | 205 | 32 (15.8) | 0.66 (0.42-1.02) |
| High | 59 | 7 (11.7) | 0.50 (0.22-1.14) |
| P for trend |  |  | 0.098 |
| **Perceived socioeconomic class** |  |  |  |
| Low | 179 | 55 (30.5) | 1.00^ |
| Intermediate | 547 | 76 (13.8) | **0.40 (0.26-0.62)** |
| High | 74 | 14 (18.4) | 0.51 (0.25-1.06) |
| P for trend |  |  | 0.073 |
| **Self-reported income** |  |  |  |
| Low | 461 | 104 (22.6) | 1.00^ |
| Intermediate | 230 | 31 (13.6) | **0.54 (0.34-0.87)** |
| High | 109 | 9 (7.9) | **0.32 (0.15-0.68)** |
| P for trend |  |  | **0.003** |
| **Smoking status** |  |  |  |
| Never smoker | 536 | 109 (20.3) | 1.00^ |
| Current smoker | 96 | 14 (15.0) | 0.69 (0.36-1.30) |
| Ex smoker | 168 | 21 (12.3) | **0.50 (0.29-0.86)** |
| **Marital Status** |  |  |  |
| Married/Cohabiting | 487 | 74 (15.1) | 1.00^ |
| Single | 43 | 6 (14.1) | 0.92 (0.37-2.30) |
| Divorced/Separated | 35 | 9 (27.0) | **2.33 (1.00-5.41)** |
| Widowed | 235 | 55 (23.4) | **1.64 (1.07-2.50)** |
| **Children 0-14 years** |  |  |  |
| No | 784 | 139 (17.8) | 1.00^ |
| Yes | 16 | 5 (27.5) | 2.98 (0.92-9.66) |
| **Children (Age categories)** |  |  |  |
| No children (0-14 years) | 784 | 139 (17.8) | 1.00^ |
| Children below 5 but not  between 6-14 years | 6 | 0 (0) | **-** |
| Children between 6-14  but not below 5 years | 10 | 5 (43.1) | **4.49 (1.23-16.48)** |
| Children below 5 and  6-14 years | 0 | - | **-** |
| **Pets** |  |  |  |
| No | 580 | 87 (15.1) | 1.00^ |
| Yes | 220 | 57 (25.7) | **1.83 (1.24-2.71)** |
| **Pets (Cats/Dogs)** |  |  |  |
| No pets | 580 | 87 (15.1) | 1.00^ |
| Cat but no dog | 92 | 14 (15.4) | 0.92 (0.49-1.72) |
| Dog but no cat | 87 | 30 (34.4) | **2.83 (1.68-4.77)** |
| Cats and dogs | 41 | 12 (30.3) | **2.45 (1.17-5.11)** |
| ° ORs were estimated using unconditional multiple logistic regression models after adjustment for sex, level of education and geographic area.  Estimates in bold are statistically significant at 0.05 level.  ^ Reference category | | | |
